# Thickness controlled proximity effects in C-type antiferromagnet/superconductor heterostructure

**DOI:** 10.1038/srep12780

**Published:** 2015-08-04

**Authors:** Awadhesh Mani, T. Geetha Kumary, J. G. Lin

**Affiliations:** 1 Center for Condensed Matter Sciences, National Taiwan University, Taipei 106, Taiwan; 2 Condensed Materials Physics Division, Materials Science Group, Indira Gandhi Centre for Atomic Research, Kalpakkam 603102, India

## Abstract

Modulation of the superconducting state possessing a C-type antiferromagnetic phase in the Nd_0.35_Sr_0.65_MnO_3_/YBa_2_Cu_3_O_7_ heterostructure is investigated, with the Nd_0.35_Sr_0.65_MnO_3_ thickness (*t*) varying from 40 to 200 nm. Both the superconducting transition temperature and the upper critical field along the *c*-axis decrease with increasing *t*; while the in-plane coherence length increases from 2.0 up to 3.6 nm. Meanwhile, the critical current density exhibits a field-independent behavior, indicating an enhanced flux pinning effect. Furthermore, low-temperature spin canting induces a breakdown and re-entrance of the superconductivity, demonstrating a dynamic completion between the superconducting pairing and the exchange field. An unexpected colossal magnetoresistance is observed below the superconducting re-entrance temperature at *t* = 200 nm, which is attributed to the dominant influence of the exchange field over the pairing energy.

Investigating the proximity effect between different ordered phases provides unique opportunities to study fundamental physics as well as new phenomena for applications^1^,^2^,^3^,^4^,^5^. The re-discovery of colossal magnetoresistance (CMR) materials^6^ and their compatibility with high-temperature superconductors (HTS)^7^ to form coherent heterostructures have further intensified the research in the area of the proximity effect^8^,^9^,^10^,^11^. In general, superconductivity is incompatible with ferromagnetism owing to the breaking apart of the Cooper pairs by the exchange field. A recent report has demonstrated the interference effects between a quasiparticle and electron in the conductance across the YBa_2_Cu_3_O_7_ (YBCO)/La_0.7_Ca_0.3_MnO_3_ interface, which establishes an understanding for the long-range proximity effects between *d*-wave superconductivity and half-metallic ferromagnetism^12^. In addition, the strength of the exchange interaction may be modulated by modifying the form of the thin film, making this process suitable for tailoring the critical parameters of superconductors for technological applications^13^,^14^. It is known that Nd_0.35_Sr_0.65_MnO_3_ (NSMO) exhibits *C*-type antiferromagnetism^15^,^16^ below the Néel temperature (*T*_*N*_ = 270 K), with spins aligned ferromagnetically along the *c*-direction but antiferromagnetically coupled within the *a*-*b* plane. However, NSMO exhibits a canted ferromagnetic (FM) phase at around 50 K via the action of the mobile electron hopping^17^. Our previous study revealed that the proximity effect of spin canting in NSMO led to a finite resistance and effective flux pinning at the superconducting state of YBCO/NSMO^10^.

In general, the proximity effect of the superconducting and antiferromagnetic (AFM) orderings is much less explored because the AFM interaction does not create an internal field significant enough to affect superconductivity. The reason NSMO has considerable effect upon YBCO is that NSMO is not an isotropic antiferromagnet (G-type) but is a C-type antiferromagnet, meaning that the Mn moment aligns parallel along the *c*-axis in one-dimensional space and compensates for the neighboring *c*-axis spin chain with opposite spins. The competition between this one-dimensional FM interaction along the *c*-axis and the two-dimensional AFM interaction in the *ab*-plane is very delicate and is therefore sensitive to temperature and/or strain, and is the reason that the canted AFM state appears at a low temperature. Hence, in this work, we use the thickness of the NSMO to effectively tune the YBCO superconducting parameters, such as the transition temperature, *T*_*c*_, the in-plane coherence length, *ξ*_ab_, and the upper critical field, *H*_*c*2_. Interestingly, we observe a change of sign in the magnetoresistance of YBCO/NSMO when NSMO reaches *t* = 200 nm, which involves a thickness-dependent competition between the exchange energy and the condensation energy of the Cooper pairs. This result demonstrates the possibility of using heterostructures of superconducting and canting phases to design a type of spintronic switch.

## Results

**Figure 1:**
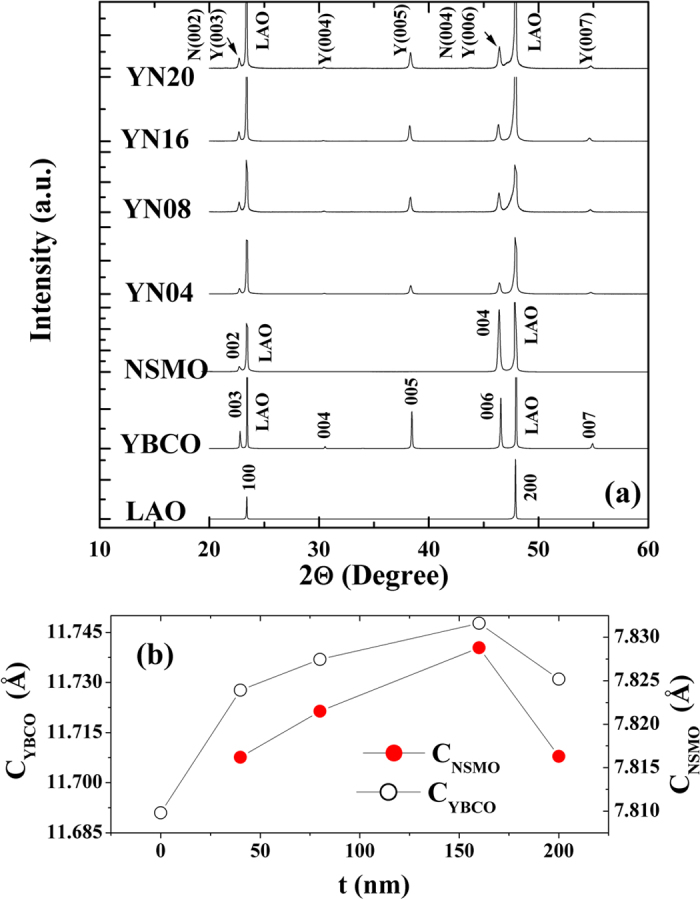
(**a**) The XRD patterns of NSMO, YBCO and various NSMO/YBCO heterostructures. Note that all the peaks are identified as *(00l).* In the index of XRD peak, N stands for NSMO and Y for YBCO. (**b**) Variations of *c*-lattice parameters of YBCO and NSMO as functions of NSMO thickness (*t*).

The X-ray diffraction (XRD) patterns of four heterostructure samples with varying NSMO thicknesses, denoted as YN04 (*t* = 40 nm), YN08 (*t* = 80 nm), YN16 (*t* = 160 nm), and YN20 (*t* = 200 nm), are shown in Fig. 1(a) along with those of single-layered NSMO and YBCO for references. The presence of only (00l) peaks in the XRD pattern suggests that the growth of the films occurs with the *c-*axis perpendicular to the substrate plane. The XRD patterns of the heterostructures match well with those of the single-layered YBCO and NSMO, indicating the phase purity and the formation of requisite structure. According to the XRD analysis, the *c*-lattice parameter for a single layer of YBCO film is 11.691 Å, which is slightly larger than the bulk values of YBCO (*c* = 11.6701 Å, *a* = 3.8820 Å, *b* = 3.8148 Å; orthorhombic structure; space group: Pmmm). This increase of the *c* parameter stems from the fact that the LaAlO_3_ (LAO) substrate induces compressive strain in the *ab*-plane of the YBCO layer because of the lattice mismatch between LAO (*a* = 3.792 Å) and YBCO. For heterostructures, the variations of the *c*-lattice parameter of NSMO (c_NSCO_) and YBCO (c_YBCO_) as functions of NSMO thickness (*t*) are shown in Fig. 1(b). The values of the *c* parameters of both NSMO and YBCO initially increase as *t* increases from 0 to 160 nm, and then decrease as *t* increases to 200 nm. Here, the value of c_YBCO_ at *t* = 0 denotes the *c*-parameter of the single-layer YBCO. The initial increase in the *c* parameter values suggests that the compressive strains in the *a-b* plane of two layers increases with the NSMO thickness, where the compression appears to attain its optimum value at *t* = 160 nm. A further increase in the thickness of the top NSMO layer leads to strain relaxation and, hence, a reduction in the *c* lattice parameter value to that found for YN20.

**Figure 2:**
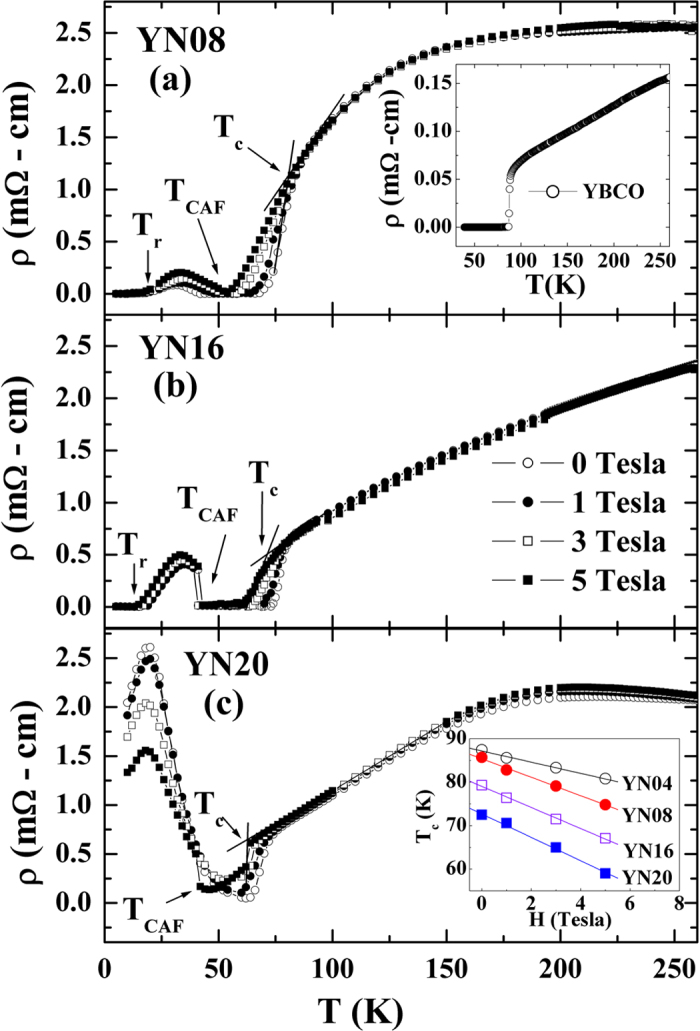
(**a**), (**b**) and (**c**) present the temperature dependent resistivity ρ(T) at various fields *H* for YN08, YN16 and YN20 respectively. The inset of (**a**) plots the ρ(T) of YBCO. Arrows indicate the positions of *T*_*r*_*, T*_*CAF*_, and *T*_*c*_. The inset of (**c**) exhibits *T*_*c*_ vs. *H* for all four samples.

**Figure 3:**
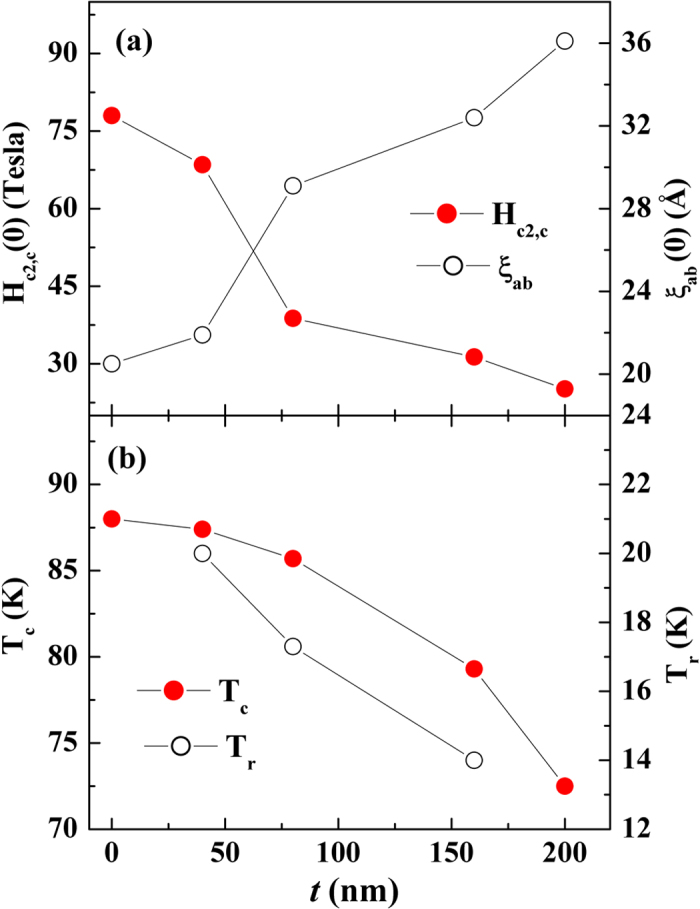
(**a**) Variations of H_c2,c_(0) and ξ_ab_(0) with *t*. (**b**) **V**ariations of *T*_*c*_ and *T*_*r*_ as functions of *t*.

The temperature-dependent resistivity, ρ(*T*), under various field strengths, *H*, from 0 to 5 T is plotted in Figs 2(a–c) for *t* = 80, 160, and 200 nm, respectively. The ρ(*T*) data for *t* = 40 nm is not shown here because they has been published previously^10^ and its temperature-dependent behavior is rather similar to that of *t* = 80 nm. The *ρ*(*T*) curves in Fig. 2 show three transitions at *T*_*c*_, *T*_*CAF*_, and *T*_*r*_, which are defined as the temperatures of the superconducting transition, spin canting, and superconductivity re-entrance, respectively. The *T*_*c*_ value is suppressed from 88 to 73 K with increasing NSMO thickness from 40 to 200 nm, indicating that the influence of the exchange field of the C-type antiferromagnet upon the superconductivity of the YBCO is still significant. The normal state resistivity of all of the heterostructure samples at 250 K is around 2.5 mΩ-cm, implying that the overall carrier concentration does not change significantly with varying *t.* Therefore, the change in *T*_*c*_ should not be related to the variation of Oxygen concentration. At temperatures below the canting transition, the maximum finite resistivity increases with the thickness, suggesting an enhancement of the vortex dissipation with increasing thickness^17^. The inset in Fig. 2(a) shows the *ρ*(*T*) data for the single-layer YBCO for comparison. The inset in Fig. 2(c) plots the *H*-dependent *T*_*c*_ for four samples, displaying a common linear trend of the *T*_*c*_ suppression under *H*, where the suppression rate (d*T*_*c*_/d*H*) changes from −1.2 to −2.7 K/T as *t* increases from 40 to 200 nm. The linear dependence of *T*_*c*_ enables us to deduce *H*_*c*2_(0) using the Werthamer–Helfand–Hohenberg formula^18^,^19^, 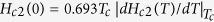. Because *H* is applied along the *c*-axis of the films, *H*_*c*2_(0) represents the value of the upper critical field along the *c*-direction. According to the Ginzburg–Landau theory for anisotropic superconductors^19^, the coherence lengths in the *ab*-plane (*ξ*_*ab*_) are related to the upper critical fields along the *c*-axis (*H*_*c*2,*c*_) with the formula 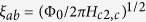. These relations are employed to deduce the values *H*_*c*2,*c*_ and *ξ*_*ab*_ for the samples under study, and the values of *H*_*c2,c*_(0) = 78 T and *ξ*_*ab*_ = 20.5 Å are obtained for the pure YBCO film, which are in good agreement with reported values^19^. The variation of *H*_*c*2,*c*_(0) and *ξ*_*ab*_ as a function of *t* is plotted in Fig. 3(a), where it can be seen that *H*_*c*2,*c*_(0) decreases while *ξ*_*ab*_ increases from 2.0 to 3.6 nm with *t* increasing from 0 to 200 nm. In Fig. 3(b), the *t*-dependent *T*_*c*_ and *T*_*r*_ are displayed, where it is clear that the *T*_*c*_ suppression does not correlate with the variation of *T*_*r*_. The value of *T*_*r*_ for *t* = 200 nm is not shown in Fig. 3(b) because finite resistivity persists even at 10 K.

**Figure 4:**
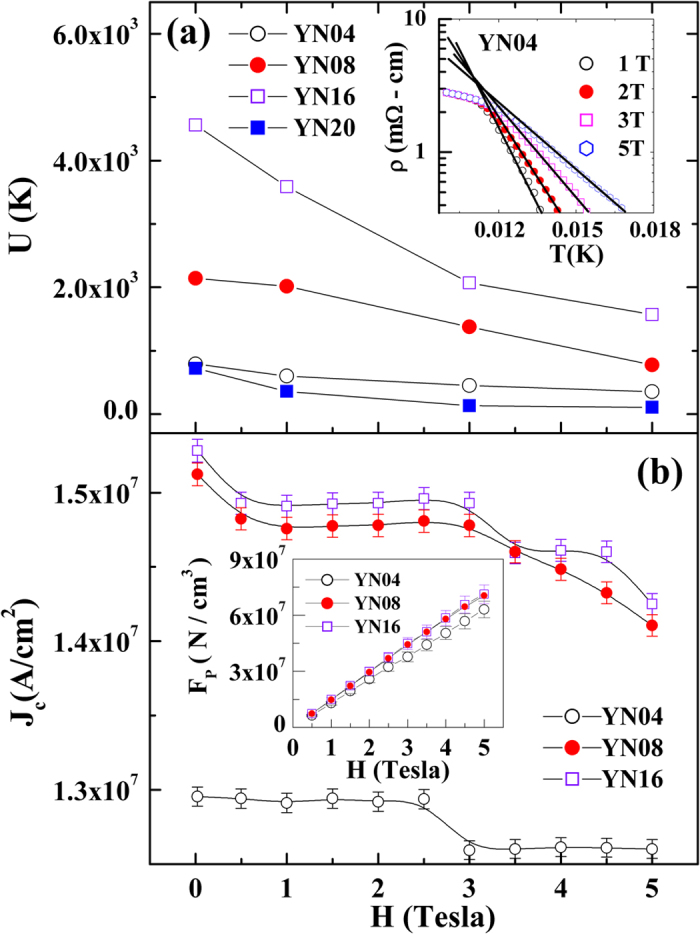
(a) Variations of pinning activation energy *U*_*0*_ of all samples with magnetic field *H*. Inset shows log (ρ) vs. 1/*T* near the resistive transition to extract *U*_*0*_. **(b)** Variation of *J*_*c*_ as a function of *H*, with inset showing the pinning force density *F*_*P*_ vs. *H* for YN04, YN8 and YN16 at 10 K.

It is known that thermally activated flux creep is very prominent in HTS at high temperatures^20^,^21^. According to the flux creep model^19^, 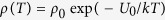, where *ρ*_0_ is the normal state resistance, *k* is the Boltzmann constant and *U*_0_ is the pinning potential. The value of *U*_0_ is a measure of the flux pinning ability of the superconductor and can be deduced from the linear fit of the log(*ρ*) vs. 1/*T* plot near the onset of *T*_*c*_ (see inset of Fig. 4a), as plotted in Fig 4(a) for all of the bilayer samples as a function of *H*. The value of *U*_0_ initially increases with *t* up to *t* = 160 nm and then decreases as *t* increases further to 200 nm. This result suggests that the pinning strength of NSMO effectively increases with its thickness up to *t* values of ~160 nm. The transport critical current density, *J*_*c*_, is measured at *T* = 10 K with *H* varying from 0 to 5 T using the standard criterion of 1 μV/cm in the current–voltage (V-I) curves. The V-I data for *t* = 200 nm exhibits a linear relation, indicating that the superconductivity is completely destroyed at 10 K. The variation of *J*_*c*_ as a function of *H* for YN04, YN08, and YN16 at 10 K is shown in Fig 4(b), where the value of *J*_*c*_ in YN16 is seen to be the largest (~1.5 × 10^7^ A/cm^2^ at *H* = 0). Remarkably, *J*_*c*_ remains nearly field independent between 0.5 and 3 T, unlike the pure YBCO film^22^. This large value of *J*_*c*_ and the sluggish field dependence are important from a technological application point of view. The macroscopic pinning force density (*F*_*P*_), calculated using *F*_*P*_ = *J*_*c*_ *×* *H*, is shown in the insets of Fig 4(b) for YN04, YN08, and YN16; where the value of *F*_*P*_ can be seen to increase monotonically with *H* for these samples, suggesting that the progressive increase of the thickness leads to an improved pinning effect and an enhanced *J*_*c*_.

## Discussion

It is noticed that the behavior of the field-dependent resistivity below the canting transition (*T*_*CAF*_) changes as *t* increases from 160 to 200 nm when we compare the low-temperature data in Figs 2(b,c). Resistivity below *T*_*CAF*_ increases with the field for the samples with *t* ≤ 160 nm (YN08 and YN16), while resistivity decreases dramatically with the field (about 50%) for *t* = 200 nm (YN20). The former behavior could be attributed to the field-enhanced vortex dissipation^10^, while the latter behavior requires a different mechanism to explain. Considering that the YBCO/NSMO heterostructure is a system where the superconducting temperature, *T*_*c*_ (~90 K), is higher than the temperature of the canting state (~40 K), the minimum energy of this system corresponds to a superconducting state with a modulated magnetic structure. Therefore, the system allows the coexistence of superconductivity and magnetism below the canting temperature. When the effective exchange energy of the canting FM state enhances with increasing NSMO thickness, the coherence length of the superconducting pair increases, as seen in Fig. 3(a). However, once the effective exchange energy exceeds the superconducting pairing energy, superconductivity disappears. Therefore, the negative magnetoresistance observed below the canting state could be regarded to be the same nature as the colossal magnetoresistance of La_0.7_Ca_0.3_MnO_3_^6^. The reason this dynamic competition has not been previously observed in the FM/superconducting system (including YBCO/LSMO) is that the FM-ordering temperature in most chosen FM materials is higher than the superconducting transition temperature. In contrast, NSMO is a unique material for this study because it is at an AFM state above the superconducting transition but is at an FM state below the transition, which allows the instant switching off of the superconductivity at the canting transition temperature. The preliminary result for a single YBCO/NSMO bilayer was published in 2009 by our group^10^. However, this present work further extends the study on the thickness dependence of proximity effect and finds a critical thickness of NSMO to stabilize the canting state at low temperature, which makes it possible to allow the resistive state remaining non-zero after the switching-off of superconductivity.

In conclusion, the temperature- and magnetic field-dependent transport properties of the YBCO/NSMO heterostructures found in this study demonstrate the effectiveness of using the NSMO layer thickness to tune the proximity of the magnetism on the superconducting parameters, such as *T*_*c*_, *J*_*c*_, and *H*_*c*2_. In particular, when the NSMO thickness is increased up to 200 nm, the exchange field of the canted state prohibits superconducting pairing, leading to a negative CMR. The new observation of switching from the superconducting to the CMR state in YBCO/NSMO may open up a new route to control oxide superconducting devices.

## Materials and methods

The YBCO (70 nm)/NSMO (*t* nm) heterostructures were deposited on a (100) LaAlO_3_ single-crystal substrate with a pulsed laser deposition system, using the deposition procedure reported in Ref. [10]. Four heterostructure samples, denoted as YN04, YN08, YN16, and YN20, were labeled with respect to their top NSMO layer thickness of *t* = 40, 80, 160, and 200 nm, respectively, where the thicknesses of the heterostructures were controlled by deposition time and later calibrated with high-resolution transmission electron microscopy (JEOL-2100F, JEOL USA Inc., USA). The phase purity of all of the films was analyzed by XRD, and the temperature- (*T*) and magnetic field- (*H*) dependent resistivity measurements were carried out on films possessing a planar dimension of 10 × 2.5 mm^2^ using the standard four-probe method with *H* perpendicular to the *ab-*plane. The V-I curves with the field varying from 0 to 5 T. Both were performed in a physical property measurement system (Quantum Design Model 6000, Quantum Design Inc., USA)).

## Additional Information

**How to cite this article**: Mani, A. *et al.* Thickness controlled proximity effects in C-type antiferromagnet/superconductor heterostructure. *Sci. Rep.* **5**, 12780; doi: 10.1038/srep12780 (2015).
